# Causes and Flock Level Risk Factors of Sheep and Goat Abortion in Three Agroecology Zones in Ethiopia

**DOI:** 10.3389/fvets.2021.615310

**Published:** 2021-01-29

**Authors:** Gezahegn Alemayehu, Gezahegne Mamo, Biruk Alemu, Hiwot Desta, Biniam Tadesse, Teferi Benti, Adane Bahiru, Muhabaw Yimana, Barbara Wieland

**Affiliations:** ^1^Animal and Human Health, International Livestock Research Institute (ILRI), Addis Ababa, Ethiopia; ^2^Department of Microbiology, Immunology and Veterinary Public Health, College of Veterinary Medicine and Agriculture, Addis Ababa University, Bishoftu, Ethiopia; ^3^College of Veterinary Medicine, Samara University, Samara, Ethiopia; ^4^National Animal Health Diagnostic and Investigation Center (NAHDIC), Sebeta, Ethiopia; ^5^Sekota Dryland Agricultural Research Center, Sekota, Ethiopia; ^6^Sekota District Livestock and Fish Development Office, Sekota, Ethiopia

**Keywords:** abortion, infectious causes, small ruminant, reproductive failures, risk factors, Ethiopia

## Abstract

A cross-sectional survey was conducted to estimate the incidence of small ruminant abortion and identify its major causes and potential risk factors in goat and sheep flocks in three agroecology and production systems of Ethiopia. Information on pregnancy outcomes and management risk factors were collected for 299 goat and 242 sheep flocks. Blood samples were collected from 133 sheep and 90 goat flocks and tested for *Coxiella burnetii, Brucella* spp., *Chlamydia abortus*, and *Toxoplasma gondii*. A causal diagram outlined relationships between potential predictor variables and abortion in the flock. The effect of management and exposure to infectious causes on the number of abortions in the flock across agroecology was tested using zero-inflated negative binomial regression. Results showed that 142 (58.68%) goats and 53 (17.73%) sheep flocks reported abortions in the 12 months before the survey. The mean annual flock abortion percentages were 16.1% (±26.23) for does and 12.6% (±23.5) for ewes. Farmers perceived infectious diseases, extreme weather conditions, feed shortage, physical traumas, and plant poisoning as the most important causes of abortion. A higher proportion of abortion was recorded during the short rainy season (March to May) and start of the short dry and cold season (June to August) in the lowland mixed crop-livestock and pastoral agroecology and production system, respectively. Overall, 65.41% sheep and 92.22% goat flocks tested positive for one or more abortion causing agents, namely, *C. burnetti, C. abortus, Brucella* spp., and *T. gondii;* mixed infection was found in 31.58% sheep and 63.33% goat flocks. Spending the night in a traditional house and providing supplementary feed for pregnant dams were important management factors which significantly (*p* ≤ 0.05) decreased the risk of abortion by 2.63 and 4.55 times, respectively. However, the presence of other livestock species and dogs in the household and exposure of the flock to *Brucella* spp. or anyone of the four tested infectious agents significantly (*p* ≤ 0.05) increased the risk of abortion in sheep and goat flocks. In general, abortion is a challenge for small ruminant production in the study area especially in lowland agroecology and calls for improvement in husbandry practices, health care and biosecurity practices.

## Introduction

Abortion is a significant problem in pregnant ewes/does and causes major financial losses for small holder livestock producers in Ethiopia. It is a limiting production factor, as it decreases the potential number of replacement stocks for flock and milk production and increases the number of unproductive females maintained for long periods in the flock (1, 2). Thus, abortion in sheep and goats significantly impacts the food security and livelihoods of rural smallholder Ethiopian farmers, as sheep and goats are an integral part of households by providing nutrition, employment and sources of income (3–5).

Causes of abortion can be broadly categorized into infectious and non-infectious causes. The commonly diagnosed infectious causes of abortion in sheep and goats are C*oxiella burnetii (C. burnetii), Chlamydia abortus (C. abortus), Brucella* spp., *Leptospira* spp., *Campylobacter fetus, Listeria* spp. *and Toxoplasma gondii (T.gondii)* (6, 7). These pathogens are also zoonotic and thus can pose serious infection risks for farming communities (8, 9). Zoonotic causes of animal abortion are prevalent and widely spread in all livestock production systems in Ethiopia (10–17). Furthermore, substantial knowledge gaps and high-risk behavioral practices toward zoonotic disease risk from livestock birth products among communities in Ethiopia increases the risk exposure to zoonotic diseases (18).

Low fertility has been reported in sheep and goats in Ethiopia (19–21). Low productivity per animal and flock offtake impact the overall contribution of sheep and goats to households in the rural areas of Ethiopia. Since the production efficiency of a flock is directly related to the number of kids and lambs produced, controlling important abortion causes increases the profitability and access to animal source food for rural poor households. Abortion in sheep and goats is often multifactorial in nature. Variation in management factors such as health care, feeding and watering practices, nutrition management for pregnant animals have an impact on survival of fetus. Moreover, production systems, seasonality and agroecological factors also significantly affect the occurrence of abortion. Furthermore, abortion management strategies through appropriate biosecurity, confirmation of the cause of abortion, prevention of common disease conditions through the use of appropriate vaccination schedules and internal parasite control programs through regular anthelmintic treatments are important to ensure the health of pregnant animals, as well as fetal survival, henceforth to reduce abortion.

Although multiple infectious causes and putative factors for abortion are identified in livestock, studies focusing on small ruminant abortions major causes and their associated risk factors are limited in Ethiopia. Some studies (15, 22) tried to address various factors under extensive production systems in specific agroecology. However, it is important to estimate the frequency of occurrence and various factors under different agroecologies and production systems to design more realistic and efficient control programmes in smallholder settings. The objectives of this study were thus to estimate the prevalence of abortion and identify the major causes and associated risk factors in small ruminants in the three agroecologies and production systems of Ethiopia.

## Materials and Methods

### Study Areas

**Figure 1:**
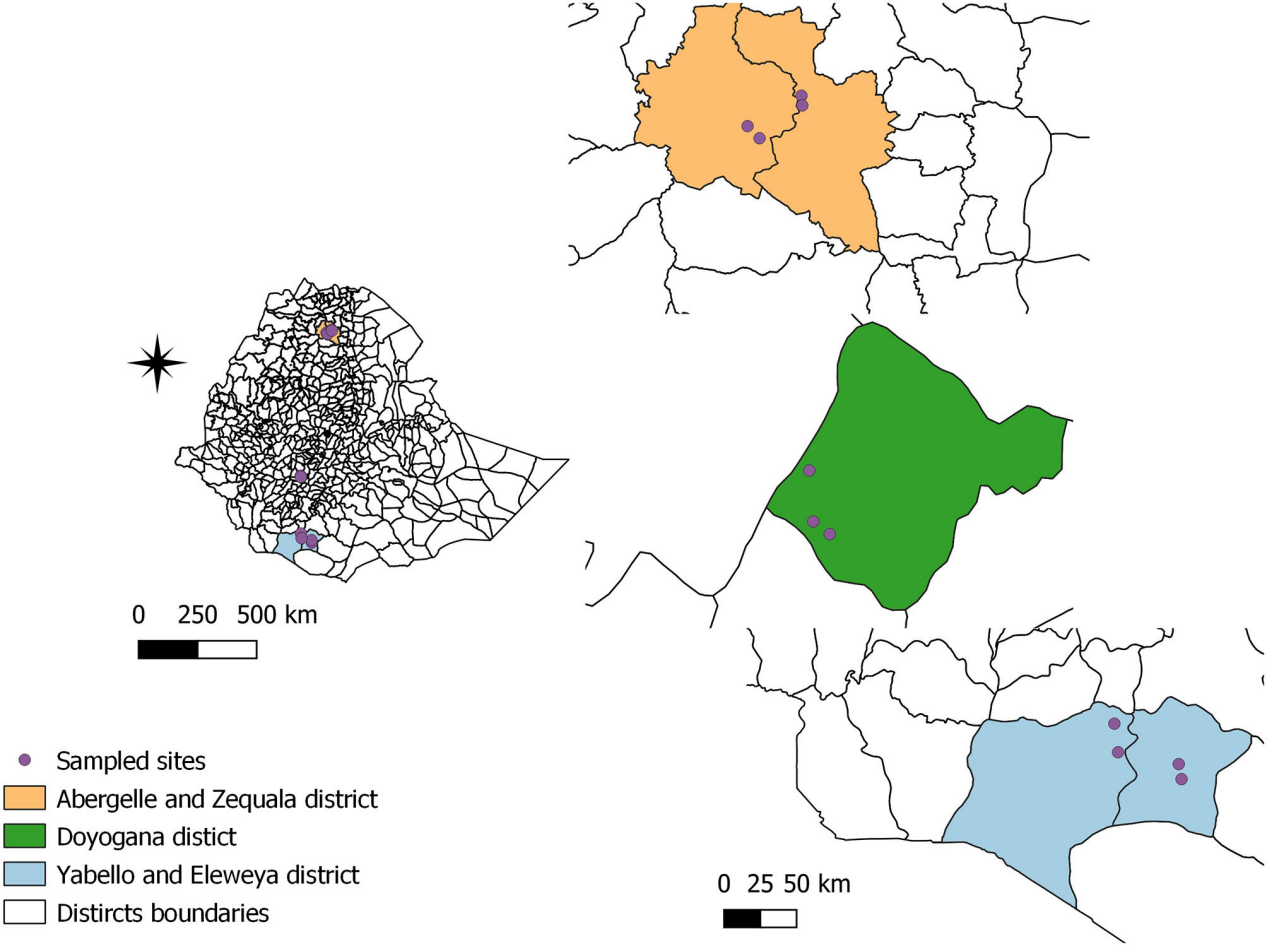
Map of study locations.

The study was conducted in 11 sites in five districts in three regional states in Ethiopia, namely, Amhara, Oromia, and Southern Nation, Nationality and People (SNNP) (Figure 1). Six of the sites were part of the CGIAR research program on Livestock (CRP Livestock) and had been selected based on agroecology and production systems, their potential for sheep and/or goat production, accessibility and willingness of the community to participate in further studies and the importance of sheep and goats to household livelihoods (23). In addition, the CRP Livestock sites were complemented by control sites from the same districts and which, in contrast to the CRP Livestock sites, had not seen any livestock interventions in recent years. The CRP Livestock interventions included improved breeding programs, better access to feed and vaccination for key diseases, and control of parasites. The agroecology and production system characteristics of the study sites are summarized in Table 1. Livestock production in Ethiopia is broadly classified into pastoral, agro-pastoral and mixed crop-livestock (MCL), peri-urban, and urban production systems (24).

**Table 1:** Description of study areas in Ethiopia.

**No**.	**District**	**Production system**	**Village**	**Altitude (masl)**	**Rainfall (mm)**	**Average temperature (^**°**^C)**
1	Abergele	Lowland, MCL	Sazba^*^	1,348	647	24
			Belteharf			
2	Ziquala	Lowland, MCL	Bilaqu^*^	1,486	732	22
			Tsitsika			
3	Yabello	Lowlands, pastoral	Derito^*^	1,588	625	20
			Dida Yabello			
4	Elwaya	Lowlands, pastoral	Adegalchet^*^	1,181	493	22
			Chari			
5	Doyogena	Highland, MCL	Ancha Sadicho^*^	2,616	1,275	15
			Hawara Arara^*^			
			Gomora Gawada			

* *Received CRP livestock interventions*.

The highland agroecology is typical for areas 2,200 m above sea level (masl) and higher in which a mixed crop-livestock production system drives the predominant economic activities. Livestock husbandry and rain-fed cropping are closely interlinked to gain complementary benefits from an optimum mixture of crop and livestock and spreading income and risks over both crop and livestock production. Natural pastures, crop residues, and crop stubbles are used as major livestock feed. Doyogana district of SNNP region represents this agroecology and production system.

The lowland agroecology with mixed crop-livestock production system denotes an elevation below 1,500 masl and is dominated by livestock production but practiced in proximity to and perhaps functional association with cropping farming. The productivity of crop farming is low in the area due to the shortage of rain. The livestock production activities are dominated by goats. The major feed resources for livestock in this production system are communal natural pastures, crop residues, crop stubbles, hay, browse plants, and weeds. Abergelle and Zequala districts in Wagihimira zone of Amhara region represented the lowland agroecology and mixed crop-livestock production system.

The lowland pastoral production system is typical for characterized by sparsely populated pastoral rangelands, where the subsistence of pastoralists is mainly based on livestock and livestock products. Livestock husbandry in this system is dominated by goats, cattle, sheep, and camels. Pastoralists in this production system take advantage of the characteristic instability of rangeland environments through strategic mobility and fencing of communal land. Yabello and Eleweya districts in Borena Zone of Oromia Region represent the lowland pastoral production system.

### Study Design and Sampling Strategies

A cross-sectional survey was conducted between July 2018 to February 2019 to collect data on the incidence of small ruminant abortion and identify its major causes and potential risk factors in goat and sheep flocks. This was complemented with a retrospective 1-year data set of pregnancy outcomes of small ruminants. After obtaining lists of households for each selected village from local field researchers, households who owned at least one sheep or goat were selected randomly using the random function in Microsoft Excel. Household heads were contacted by facilitators and asked for their willingness to participate and to plan the timing of the interview. Only one person per household, whether male or female, was interviewed with the aim to have 50% female respondents if possible. Assuming a standard error (SE) of 2.8%, the required sample size for respondents of the household questionnaire interviews was calculated using the formula [*n* = 0.25/(SE^2^)] given by Arsham (25) with 95% confidence level. Accordingly, a total of 318 respondents were required for house-to-house interviews, resulting in 64 households per district.

The blood samples used to determine the serostatus of antibodies against abortive pathogens in the flock were collected from flocks of randomly selected households who participated in the interview. Due to financial and logistical limitation, diseases were prioritized according their likely burden on small ruminant and human population in Ethiopia and availability of diagnostic kits in Ethiopia. Accordingly, six abortion causing agents (*C. burnetii, C. abortus, Brucella* spp., *Leptospira* spp*., T. gondii*, and *Neospora caninum)* were selected to be included in this study based on existing literature and expert opinion. Unfortunately, we were not able to source the diagnostic kits for *Leptospira* spp. and *Neospora caninum*. Therefore, we only included *C. burnetii, C. abortus, Brucella* spp., and *T. gondi* for which we could the analysis in this paper. Sample size was calculated using web-based Epidemiological Calculators (26) with the following predetermined parameters: 50% of the expected individual prevalence of each pathogen, a confidence level (CL) of 95%, and a desired level of precision (d) of 5%. Since animals within the same households tend to have more similar outcomes, the total sample size was adjusted for clustering at the household level using the formula described by Dohoo et al. (27):

$$\begin{array}{l} n\prime =n(1+p(m-1)) \end{array}$$

where

*n*′ is the new sample size,
*n* is the original sample size estimate,
*p* is the intra-cluster correlation coefficient
*m* is the number of ewes /does sampled per flock.

The intra-cluster correlation coefficient (*P*) for the majority of infectious diseases is usually between 0.05 and 0.2 (28). Accordingly, a value of 0.2 was taken for the initial sample size calculation. We planned to sample an average of 8 animals (5 goats and 3 sheep) from each household. Hence, a minimum of 845 small ruminants were required for study. However, the sample size was increased to 1,226 to allow for poor quality samples, drop-outs and to increase precision. Accordingly, a total of 154 households were targeted for blood sample collection from their animals.

### Data Collection Processes and Tools

The data were collected through a face-to-face structured questionnaire interview. The questionnaire interviews were conducted by four trained veterinarians and/or animal production experts from the National Agricultural Research system (NARS) who spoke the local language of the respective study site. The participants were informed that the aim of the survey was to get information on small ruminant abortions. The questions were designed based on a literature review and the experiences of the researchers and pretested with 15 farmers who were not included in the study population. After necessary adjustments, questions were coded using Epi Info™ 7.2.1.0 software and copied on mobile tablet devices for digital data collection.

Questions addressed pregnancy outcomes of goats and sheep over 1 year prior to the interview, season of the year, abortion strikes in the flock, perceived causes of small ruminant abortion and flock management practices. Data on management related potential factors included household demographics data (sex, age, and educational level of household head, location), livestock keeping type (mixed crop livestock pastoral), flock type and structure (small ruminant species, flock type, flock size, and presence of other livestock in the households), feeding and husbandry (grazing land, housing type, confinement level, source of water, distance travel to grazing pasture), management of pregnant dam approaching delivery (supplementary feeding, housing), breeding management (breeding buck/ram ownership, buck/ram stay in the flock), biosecurity practices (routine manure cleaning, birth products disposal practice, action on frequently aborting dam, dog and cat access to the flock) and CRP livestock intervention status.

During the interview, respondents were asked about their confidence in the estimates provided in retrospective information. If they were not confident about their estimates, the data entry for that question was left empty and treated as missing. Moreover, information collected through the questionnaire interviews about the number of pregnant animals delivered and aborted was matched with data collected longitudinally on recorded sheets by recruited enumerators in the households belonging to intervention sites. In cases where information did not match, the flock owners and data enumerators were approached to validate the discrepancy and the data were corrected wherever possible.

### Serum Sample Collection and Processing

About 6–8 ml blood was collected from the jugular vein into 10 ml sterile plain vacutainer tubes. Individual animal biodata was gathered during sample collection. The tubes were then labeled with a unique identification number and kept protected from direct sun light. The samples were placed in the slant position until the blood was clotted and sera were separated. The sera were separated from clotted blood after centrifugation at 1,500 g for 10 min at Yabello Pastoral and Dryland Agriculture Research Center, Sekota Dry land Agriculture Research Center, and Wolayita Sodo Regional Veterinary Laboratory. The serum was transferred into a sterile cryovial tube bearing the identification number and transported to the National Animal Health Diagnostic and Investigation Center (NAHDIC), Sebeta, Ethiopia, for laboratory analysis. The samples were transported to the laboratory at +4 using a portable fridge, plugged into a car, and then stored at −20°C until analyzed.

### Laboratory Analyses

Commercial enzyme-linked immunosorbent assays (ELISA) were used to detect antibodies against *C. burnetii, Brucellas* spp. and for Toxoplasma*., C. abortus* and *T. gondii* at NAHDIC. For *C. burnetti* the Antibody Test Kit, (IDEXX® Switzerland AG, CH-3097 Liebefeld-Bern Switzerland), for Chlamydia, the *C. abortus* Antibody Test Kit (IDEXX® Switzerland AG, 3097 Liebefeld-Bern Switzerland), for *Brucella* spp., Svanovir TM Brucella-Ab c-ELISA test kits (Svanova Biotech, Uppsala, Sweden) and for Toxoplasma the *T.gondii* Antibody Test Kit, (IDEXX® Switzerland AG, 3097 Liebefeld-Bern Switzerland) were used. Test procedure, computation of the sample to positive rations, and final interpretation of the results were performed following the protocols provided by the respective kit manufacturers. Briefly, sera samples, negative and positive controls were diluted at 1:400 using wash solution. One hundred μL of pre-diluted negative and positive control and samples were added into microtiter plate and incubated for 1 h at 37°C. The plate was then washed 3 times and 100 μL of conjugate was added to each well. Plate were incubated at +37°C for 1 h after the microplate was sealed using plate covers to avoid any evaporation. The plates were agian washed 3 times. Finally, 100 μL of substrate was added to each well and incubated at 18–26°C for 15 min. Then 100 μL of stop solution was added to each well and the result was read at a wavelength of 450 nm. The OD of the positive control (PCx̄) and the OD of the samples (sample A_450_) are corrected by subtracting the OD of the negative control (NCx̄). Sample to positive ratio (S/P%) was computed as $\frac{100\times sample\;A450-NC\bar{x}\;}{\;PC\bar{x}\;-\;NC\bar{x}\;}$. An animal was considered to be infected when the serum presented an S/P% ≥ 30 for *Brucellas* spp., ≥40 for *C. burnetii*, and *C. abortus* and ≥50% for *T. gondii*.

### Data Management and Analyses

The recorded responses from questionnaire interviews were transferred and stored on a personal laptop computer and subsequently exported to Microsoft Excel where data cleaning and integration were undertaken. Laboratory results were entered into Microsoft Excel version 15 and linked to the respective household data. The data were transformed to create flock level tables in the database. The variables created from the laboratory data were crossmatched and combined with the questionnaire data. The data cleaning and statistical analyses were conducted in STATA 15.1 (Stata SE/15.1, Stata Corp., College Station, TX, USA).

The unit of analysis was the flock. A flock was defined in this study as sheep and goats owned by a household. The outcome of interest was the number of abortion cases in a sheep and goat flock. Annual abortion percentage were calculated as lamb/kids lost before the expected date of parturition divided by pregnant ewes/dose in the flock for 1 year multiplied by hundreds. Descriptive statistics such as mean, standard deviation (SD) were used to describe the number and percentage of abortion. Cross tabulation and frequency tables were used to describe proportions.

Unconditional association between potential risk factors and outcomes of interest was tested in univariable models. The maximum likelihood method was used to estimate parameters describing the relationship between predictor variables and outcomes of interest. Incidence rate ratio (IRR) was used to measure the effect of various predictors on the outcomes of interest. The Poisson distribution has been considered in the context of regression analysis for describing count data where the sample mean, and sample variance are almost equal. The data was considered overdispersal if the sample variance was significantly greater than the sample mean (29). Since the count of abortions in this study showed overdispersion, the negative binomial regression has been found to fit our data well. Moreover, Zero-Inflated models have been used for modeling the count data set, which showed a large proportion of zeros. It was assumed that the excess zeros in our dataset were from two sources, either there was no pregnancy or no abortion in the flock. The model goodness of fit was examined by likelihood using the Aikake Information Criteria (AIC) and the Bayesian Information Criteria (BIC). The likelihood ratio test was used to compare between negative binomial and zero-inflated negative binomial regression models. Models with smaller values of AIC and BIC were considered to fit the data. A random effects regression model was used to account for the clustering of flocks within villages. Variables showing an unconditional association with the outcome were selected for multivariable analysis, using *p* < 0.15 as the criterion for inclusion.

**Figure 2:**
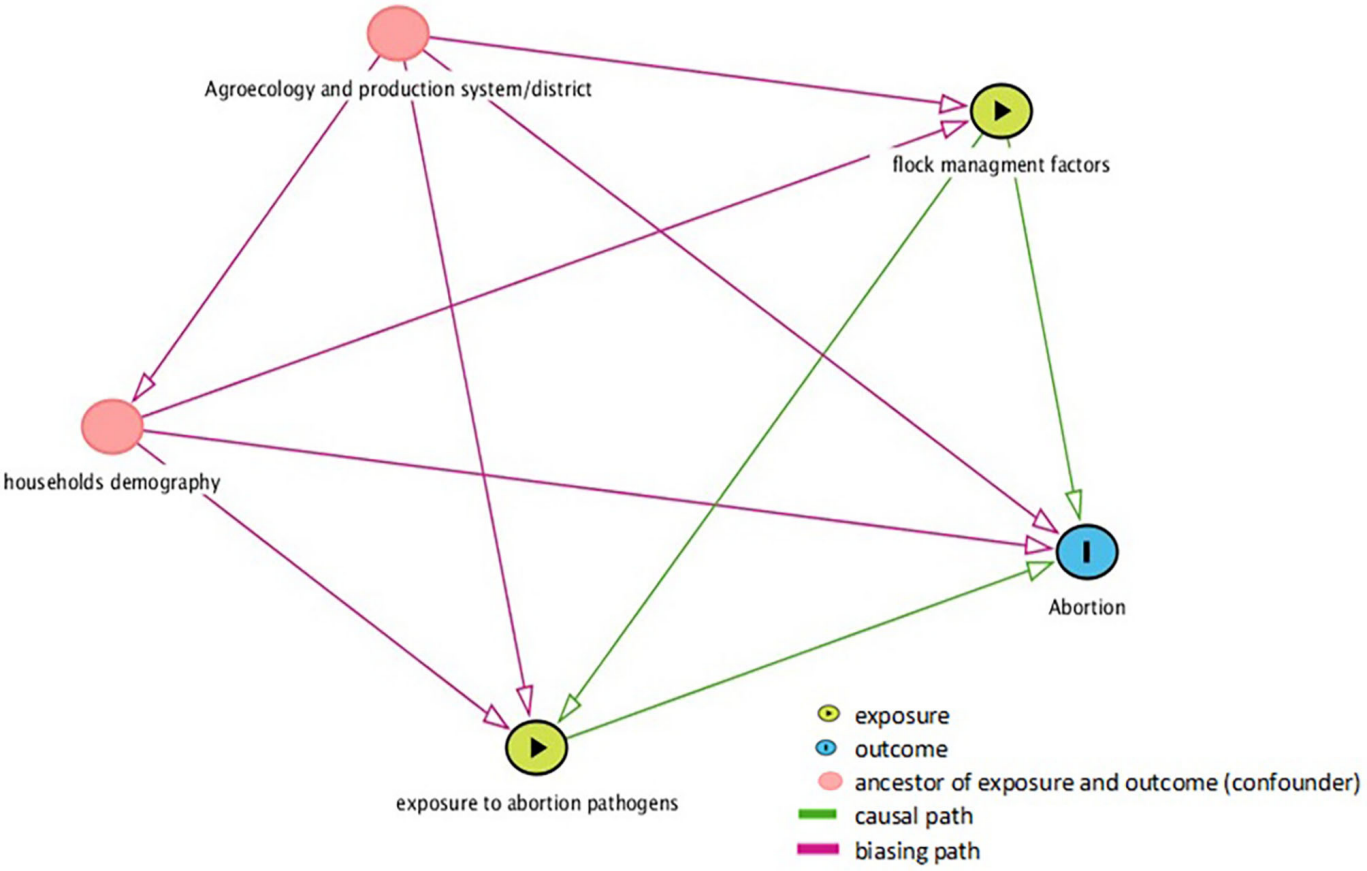
Causal diagram generated in DAGitty (30) postulating the relationships between the potential predictors and small ruminant abortion sampled in three agroecology and production system of Ethiopia.

A causal diagram was generated in the browser-based environment DAGitty® (30) to identify causal relationships between potential predictors and abortion in the flock (Figure 2). This diagram was used to identify plausible predictors of abortion in the flock and intervening variables (the variable lies along the path between the exposure and the outcome of interest) and potential confounders (variables antecedent to the exposure variable). Multivariable models were built with number of abortions and the main factor (s) of interest related to management factors and exposure to abortion causing agents. Variables in the causal diagram, which could be potential confounders, were retained in the model if the parameter estimates of any explanatory variable changed more than 20%. Variables between the exposure variables and outcome variables were excluded from the model as they were intervening variables. Plausible biological interactions between variables were evaluated and included if significant. The likelihood ratio test was used to evaluate the significance of variables. *P* ≤ 0.05 was considered significant.

## Results

**Figure 3:**
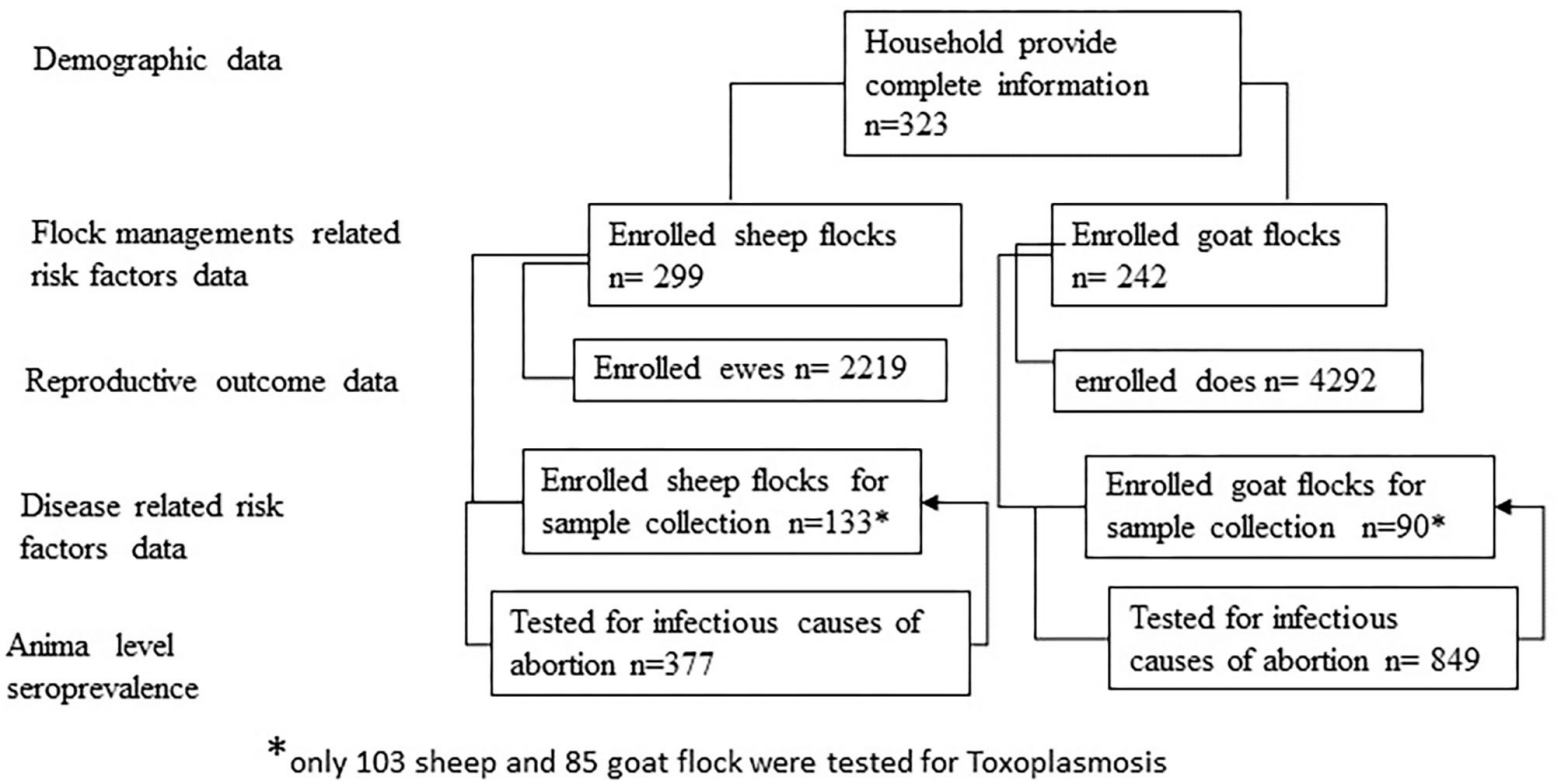
Study subject enrolment flow diagram.

Information on pregnancy outcomes and managemental risk factors was collected from a total of 299 goat and 242 sheep flocks. Blood samples were collected from a total of 1,226 animals from 223 (133 sheep and 90 goat) flocks and were tested for *C. burnetii, Brucellas* spp., and *C. abortus* and 994 samples from 192 (103 sheep and 85 goat) flocks were tested for *T. gondii*. Figure 3 shows the study subject enrolment flow.

### Flocks Enrolled in the Study

From the total 242 goat flocks enrolled in the study, 114 (47.11%), and 128 (52.89%) were managed under a lowland MCL and pastoral agroecology, respectively. Whereas, from the total 299 sheep flocks, 107 (35.79%), 126 (42.14%), and 66 (22.07%) were lowland MCL, pastoral, and highland MCL agroecology and production systems, respectively.

Long-eared Somali and Abergele were the predominant goat breeds kept by pastoralists in the lowland Borena zone and the lowland of Waghimira, respectively. Adilo, Sekota and Black head Somali and are the predominant sheep breeds kept in Doyogena district, Waghimira, and Borena zones, respectively. The mean (±s.d.) flock size of goats and sheep was 30.19 (±25.36, median = 13) and 13.6 (±13.4, median = 5) animals, respectively. The majority of the flocks were mixed flocks with both sheep and goats (81.33%), while 14.23% and 4.44% were sheep-only and goat-only flocks, respectively. Flocks were kept under traditional extensive management systems and therefore fully dependent on grazing land, with overall limited input. The majority of the flocks (89.28%) were grazed freely on pastures during daytime while few flocks were tethered (0.74%). About 41.59% of small ruminant flocks spent the night in an open enclosure, while 58.41% of the flocks spent the night in the traditional shoat house. All day-to-day herding decisions were made by the owner and breeding was uncontrolled. Ewes/does in 84.84% of the flocks were mainly mounted by rams/bucks from the same flock, while 15.16% of the flocks utilized rams/bucks from other flocks. Fertile bucks/rams were remained continuously with a group of females throughout the year.

### Estimation of Sheep and Goat Abortion

**Figure 4:**
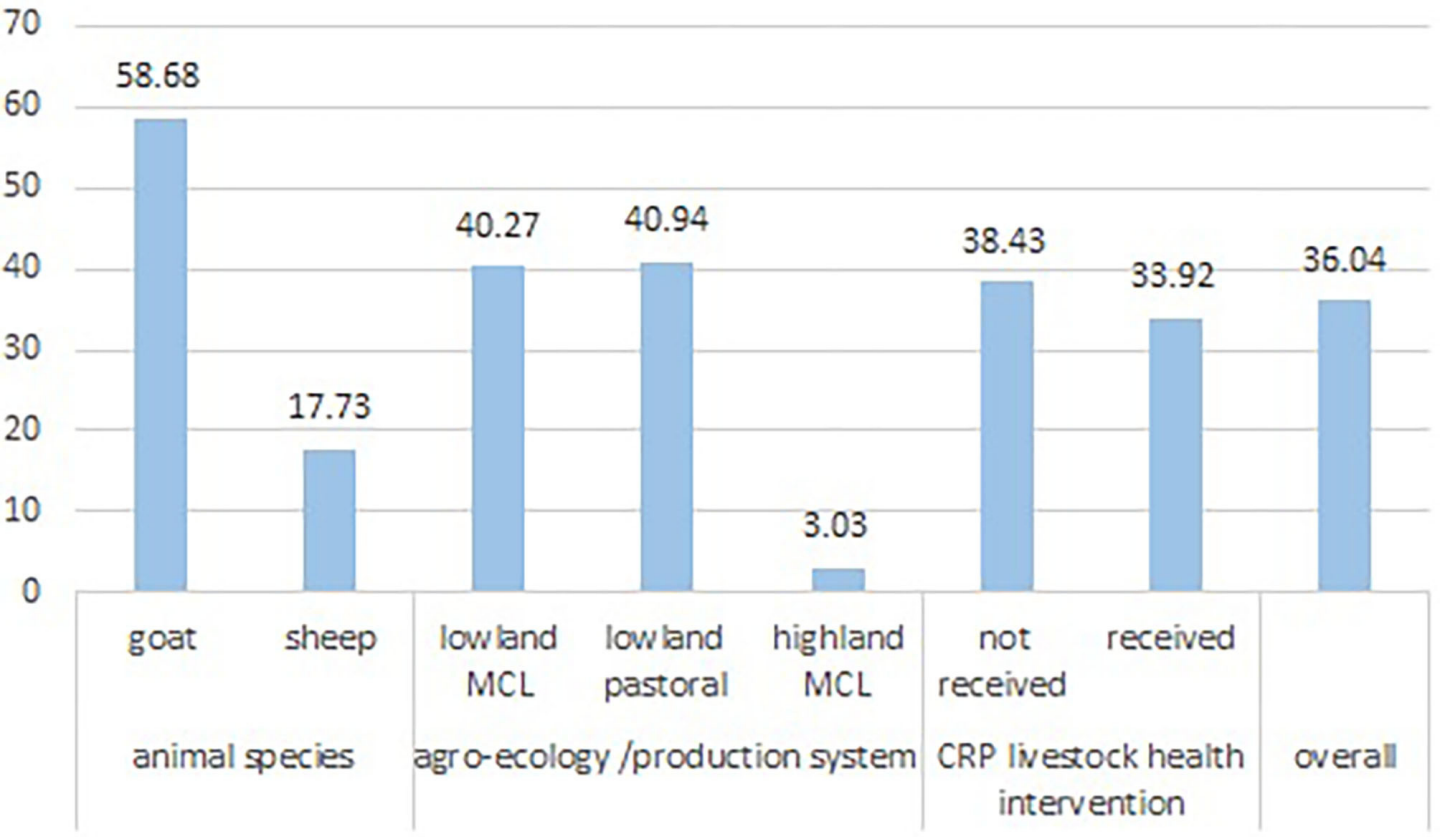
Proportion of flock with abortion in sheep and goat in three agroecology and production system of Ethiopia.

Of the 242 goat and 299 sheep flocks observed, 142 (58.68%) of goat and 53 (17.73%) sheep flocks reported abortions in the 12 months before the study. Occurrence of at least one abortion in the flock was significantly higher (*P* = 0.000) in goat flocks (142/242, 58.68%) than sheep flocks (53 of 299, 17.73%). Overall, very few numbers of flocks from highland mixed crop livestock production system (2 of 66, 3%) reported abortions, which was significantly (*P* = 0.00) lower than other production systems. However, comparable proportions of flocks were affected in the lowland mixed crop- livestock production system (40.27%) and the lowland pastoral production system (40.94%) (Figure 4).

A total of 860 does and 153 ewes abortion cases were recorded from 4,995 pregnancies, of 3,380 does and 1,615 ewes monitored over 1 year, respectively. On average, 3.55 does and 0.5 ewes aborted per flock. The annual number of abortions per flock ranged from 0 to 60 (median = 1) and 0–12 (median = 0) for the sheep flocks. The mean annual flock abortion percentages were 16.1% (±26.23) for does and 12.6% (±23.5) for ewes. Annual flock abortion percentage was higher (*p* < 0.05) among small ruminant flocks in lowland mixed crop-livestock production system than highland mixed crop, livestock, and lowland pastoral production systems. Table 2 shows the annual abortion percentage in three agroecology and production systems aggregated by small ruminant species.

**Table 2:** Mean annual sheep and goat abortions percentage in three Agroecology and production system of Ethiopia.

**Ago-ecology and production**	**Species**	**No. of flock examined**	**No. at risk**	**No. aborted (mean)**	**Abortion percentage (%) (±SD)**
Overall		541	4,995	1,013 (1.9)	14.15 (24.78)
Lowland MCL	Goat	114	1,682	380 (3.33)	22.23 (27.71)
	Sheep	102	643	72 (0.67)	21.20 (26.19)
Lowland Pastoral	Goat	128	1,698	480 (3.75)	10.52 (23.56)^**^
	Sheep	123	828	79 (0.63)	12.01 (24.25)8^*^
Highland MCL	Sheep	65	144	2 (0.03)	0.00 (0.00)^**^
Total	Goat	242	3,380	860 (3.55)	16.11 (26.23)
	Sheep	290	1,615	153 (0.51)	12.55 (23.45)

* *Significant at p ≤ 0.01*,

** p ≤ 0.001, SD = standard deviation.

### Perceived Causes of Small Ruminant Abortion

Abortion was called “Koyisayech” by the Agew community in the lowland of Waghimira zone, “Salesa” by Borena pastoralists of the Oromo community in lowland Borena, and “Kara” by the Kembata community in the highland of Doyogana. Goat was considered as the most affected livestock species by abortion by 99.2 and 98.4% of respondents in lowland MCL and pastoral production system, respectively. Of the 1,011 abortion cases recorded in the lowland MCL and pastoral agroecology and production system, animal owners recognized only the causes of 509 (50.34%) abortion cases during the individual interview. From the recognized causes, extreme weather conditions (30.21%), disease (26.89%), and feed shortage (25.68%) were the first, second, and third most important causes of abortion in lowland MCL agroecology and production system, respectively. Nevertheless, in lowland pastoral agroecology and production system, disease (56.74%) was considered the major cause, followed by feed shortage (17.42%) and plant poisoning (14.61%) (Table 3). The two abortion cases in highland MCL agroecology and production system were caused by physical trauma as perceived by the farmers.

**Table 3:** Perceived causes of sheep and goat abortion in lowland MCL and pastoral agroecology and production system in Ethiopia.

**Cause of abortion**	**Relative contribution of causes, proportion, n (%)**		***P*-value**
	**Lowland MCL** ** (*n* = 331)**	**Lowland pastoral** ** (*n* = 178)**	
Infectious diseases	89 (26.89)	101 (56.74)	0.000
Extreme weather condition	100 (30.21)	15 (8.43)	0.034
Feed shortage	85 (25.68)	31 (17.42)	0.000
Physical traumas	53 (16.01)	5 (2.81)	0.000
Plant poisoning	4 (1.21)	26 (14.61)	0.000

MCL, Mixed crop livestock.

**Figure 5:**
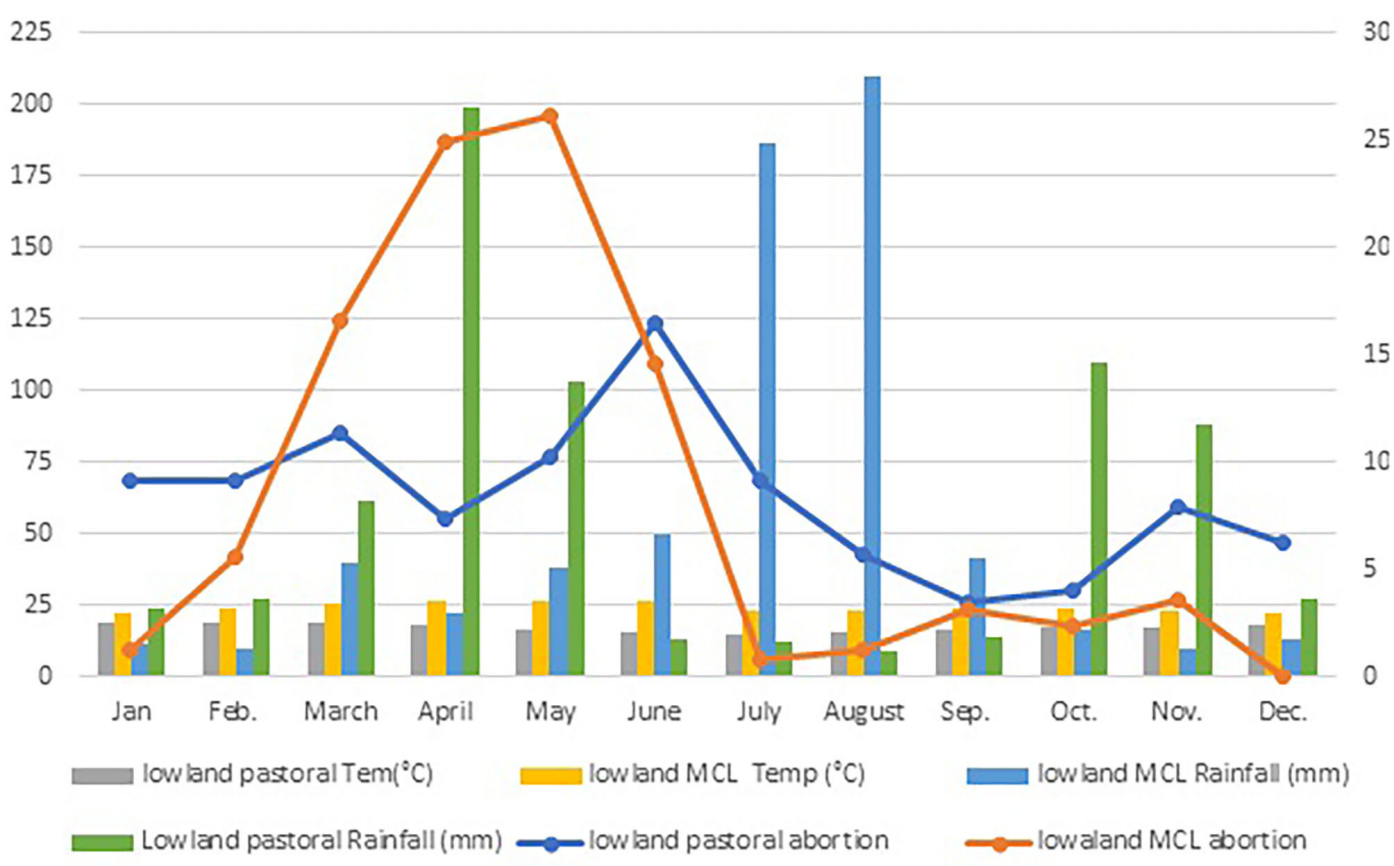
Monthly levels of sheep and goat abortion in comparison with mean monthly temperature and rainfall distribution for lowland MCL and pastoral agroecology and production system.

Monthly incidence of abortion in sheep and goat flocks in dryland ecosystem is presented in Figure 5. A higher proportion of abortion was found in the lowland MCL agroecology and production system during the short rainy season-*Belg* (March to May). The abortion numbers peaked in May, the hottest month of the year. However, in lowland pastoral agroecology and production system, higher proportions of abortions were found to occur at the start of the short dry and cold season -*Adoolessa* (June to August) followed by the long dry season-*Bona Hagayya* (December–February).

### Flock Level Seroprevalence of Major Infectious Causes of Abortion

Overall, 76.23% sheep and goat flocks tested positive for one or more abortion causing agents, namely, *C. burnetti, C. abortus, Brucella* spp., and *T. gondii;* mixed infection was found in 44.39% of the 223 flocks tested. Ninety six (43.05%), 73 (32.74%), and 57 (25.56%) flocks tested positive to *C. burnetti, C. abortus*, and *Brucella* spp., respectively. *Toxoplasma gondii* infection was detected in 91(47.4%) of the 192 flocks tested. Details, including statistically significant differences across the small ruminant species flocks and agroecology and production system are presented in Table 4.

**Table 4:** Flock level seroprevalence of major infectious causes abortion in small ruminant in three agroecology and production systems of Ethiopia.

	**Prevalence estimates (%) (calculated at** ***p*** **=** **0.05, CI** **=** **0.95)**						
**Category**	**Variable**	***C. burnetii*** ** (n = 223)**	***C. abortus*** ** (*n* = 223)**	***Brucella* Spp.** ** (n = 223)**	***T.gondii*** ** (*n* = 192)**	**at least one infection** ** (*n* = 223)**	**Mixed infection** ** (*n* = 223)**
Flock type	Goat	73.3 (64.0, 82.6)	42.2 (31.8, 52.6)	24.4 (15.3, 33.4)	48.2 (37.4, 59.1)	92.2(86.6, 97.9)	63.3 (53.2, 73.45)
	Sheep	22.6 (15.4, 29.8)^**^	26.3 (18.7, 33.9)^*^	26.3 (18.7, 33.9)	46.7(37.1, 56.3)	65.4 (57.2, 73.6)^**^	31.6 (23.6, 39.6)^**^
Production system	Lowland MCL	62.9 (50.5, 75.3)	37.1 (24.7, 49.5)	6.45 (0.2, 12.7)	31.6 (19.1, 44.0)^**^	70.9 (59.3, 82.6)	46.8(34.0, 59.5)
	Lowland pastoral	57.6 (47.7, 67.5)^**^	26.3 (17.4, 35.1)	24.2 (15.7, 32.8)^**^	57.6 (47.6, 67.5)	79.8 (71.7, 87.8)	50.5(40.5, 60.5)
	Highland MCL	0	38.7 (26.2, 51.1)	46.7 (33.9,59.5)	44.4 (27.3, 61.5)	75.8 (64.8, 86.8)	32.3(20.3, 44.2)
Overall		43.1 (36.5, 49.6)	32.7 (26.5, 38.9)	25.5 (19.7, 31.3)	47.4 (40.2, 54.5)	76.2 (70.6, 81.9)	44.4 (37.8, 50.9)

* *Significant at p ≤ 0.05*,

** *p ≤ 0.001*.

### Factors Affecting Number of Abortions in Sheep and Goat Flocks

The effect of management and exposure to infectious causes on the number of abortion in the flock across agroecologies was tested using univariate zero-inflated negative binomial regression. The casual diagrams (Figure 2) helped to describe the postulated links between sheep and goat abortion and potential predictors. The diagram illustrates that sheep and goat management practices as a group (flock type and structure, feeding and watering, housing, pregnant dam management, breeding management, biosecurity practices, and herd health intervention) and exposure to abortion pathogens are direct exposure variables associated with abortion. The demographic factors of household head, district and agroecology and production system were assumed to be potentially confounding variables, associated with both exposure and outcome variables, but not a consequence of exposure to it. However, the district was removed from the model due to the multicollinearity effect of the production system. Unconditional association between potential predictors and the occurrence of abortion in the flock is presented in Table 5.

**Table 5:** List of all predictors relating to small ruminant management practice, household demography descriptions, exposure to abortion pathogens and unconditional association with history of abortion and number of abortions.

**Viable**	**Category**	**No. flock observed**	**No. flock affected**	**Odds Ratio**	**95% CI**	***P*-value**	**IRR**	**95% CI**	***P*-value**
Agro- ecology and production system	Lowland MCL	221	89 (40.27)	1			1		
	Lowland Pastoral	254	104 (40.94)	1.03	0.71, 1.48	0.88	1.08	0.75, 1.56	0.66
	Highland MCL	66	2 (3.03)	0.05	0.01, 0.19	0.00	0.05	0.01, 0.29	0.00
District	Abergele	112	49 (43.75)	1			1		
	Doyogena	66	2 (3.03)	0.04	0.01, 0.17	0.00	0.05	0.01, 0.30	0.00
	Eleweya	152	70 (46.05)	1.10	0.67, 1.79	0.71	1.27	0.80, 2.04	0.32
	Yabello	102	34 (33.33)	0.64	0.37, 1.12	0.12	0.79	0.46, 1.36	0.40
	Ziquala	109	40 (36.7)	0.75	0.43, 1.28	0.29	1.01	0.59, 1.74	0.96
Sex of household head	Female	61	18 (29.51)	1			1		
	Male	480	177 (36.88)	1.40	0.78, 2.49	0.26	1.90	0.96, 3.78	0.07
Age of household head	<30	185	66 (35.68)	1			1		
	30–60	324	119 (36.73)	1.05	0.72, 1.52	0.81	0.80	0.54, 1.17	0.25
	>60	32	10 (31.25)	0.82	0.37, 1.83	0.63	0.67	0.29, 1.52	0.34
Education level household head	None	402	164 (40.8)	1			1		
	Primary	98	22 (22.45)	0.42	0.25, 0.70	0.00	1.00	0.57, 1.74	1.00
	Secondary and above	41	9 (21.95)	0.41	0.19, 0.88	0.02	0.84	0.34, 2.11	0.72
Flock type	Goat	242	142 (58.68)	1			1		
	Sheep	299	53 (17.73)	0.15	0.10, 0.22	0.00	0.25	0.17, 0.36	0.00
Small ruminant flock mix	Goat only	24	9 (37.5)	1			1		
	Sheep only	77	4 (5.19)	0.09	0.02, 0.34	0.00	0.33	0.08, 1.28	0.11
	Mixed	440	182 (41.36)	1.18	0.50, 2.74	0.71	1.51	0.65, 3.50	0.34
Presence of other livestock	Small ruminant only	17	2 (11.76)	1			1		
	≤ 2 other livestock species	134	40 (29.85)	3.19	0.70, 14.61	0.14	2.17	0.34, 13.7	0.41
	>2 other livestock species	390	153 (39.23)	4.84	1.09, 21.47	0.04	3.81	0.62, 23.3	0.15
Flock size	<15	351	80 (22.79)	1			1		
	15, 30	110	60 (54.55)	4.07	2.59, 6.38	0.00	1.93	1.25, 2.99	0.00
	>30	80	55 (68.75)	7.45	4.37, 12.72	0.00	5.40	3.39, 8.62	0.00
Grazing land	Communal and private	113	41 (36.28)	1			1		
	Communal only	374	147 (39.30)	1.14	0.74, 1.76	0.56	1.99	1.29, 3.06	0.00
	Privately owned only	54	7 (12.96)	0.26	0.11, 0.63	0.00	4.64	1.64, 13.16	0.00
Distance to grazing land	<2 km	215	58 (26.98)	1			1		
	2–4 km	219	92 (42.01)	1.96	1.31, 2.94	0.00	1.36	0.90, 2.06	0.15
	>4	107	45 (42.06)	1.96	1.21, 3.20	0.01	2.14	1.30, 3.52	0.00
Source of water	Liver/spring	255	89 (34.9)	1			1		
	Tab water	19	0 (0)				0(0)		
	Stagnant	264	105 (39.77)	1.23	0.86, 1.76	0.25	1.13	1.63, 0.5	0.78
Feeding system	Free grazing	483	194 (40.17)	1			1		
	Tethered	4	0 (0)				0(0)		
	Both	54	1 (1.85)	0.03	0.00, 0.20	0.00	0.03	0.00, 0.30	0.00
Supplementary feed for pregnant	No	447	178 (39.82)	1			1		
	Yes	94	17 (18.09)	0.33	0.19, 0.58	0.00	0.33	0.18, 0.60	0.00
Separate house for pregnant	No	399	157 (39.35)	1			1		
	Yes	142	38 (26.76)	0.56	0.37, 0.86	0.01	0.84	0.53, 1.32	0.45
Breeding male ownership	No	82	26 (31.71)	1			1		
	Yes	459	169 (36.82)	1.26	0.76, 2.07	0.38	1.40	0.79, 2.46	0.25
Ram/buck stay in the flock	>2	62	7 (11.29)						
	2–4	199	80 (40.2)	5.28	2.29, 12.19	0.00	0.44	0.17, 1.09	0.08
	>4	280	108 (38.57)	4.93	2.17, 11.23	0.00	0.39	0.16, 0.97	0.04
CRP livestock health intervention	Not received	255	98 (38.43)	1			1		
	Received	286	97 (33.92)	0.82	0.58, 1.17	0.28	1.27	0.89, 1.83	0.19
Housing/shelter at night	None shelter paddock	225	102 (45.33)	1			1		
	Traditional house	316	93 (29.43)	0.50	0.35, 0.72	0.00	0.48	0.33, 0.68	0.00
Routine manure cleaning	No	166	74 (44.58)	1			1		
	Yes	375	121(32.27)	0.59	0.01, 0.41	0.86	0.63	0.43, 0.92	0.02
Birth products disposal practice	Bury/burn	44	2 (4.55)	1			1		
	Feed to dog	379	157 (41.42)	14.85	0.00, 3.54	62.26	17.22	3.13, 94.80	0.00
	Disposed to environment	118	36 (30.51)	9.22	0.00, 2.12	40.16	22.83	4.02, 129.7	0.00
Action on frequently aborting dam	Keep in the flock	132	62 (46.97)	1			1		
	Sell	267	101 (37.83)	0.69	0.08, 0.45	1.05	0.64	0.43, 0.95	0.03
	Slaughter	36	21 (39.00)	1.58	0.23, 0.75	3.33	1.15	0.58, 2.28	0.69
Dog access to the flock	Not at all	152	39 (25.66)	1			1		
	Yes, neighbor dog	139	54 (38.85)	1.84	0.02, 1.12	3.03	2.11	1.21, 3.67	0.01
	Yes, own dog	239	99 (41.42)	2.05	0.00, 1.31	3.20	2.49	1.53, 4.06	0.00
Cat access to the flock	Not at all	211	90 (42.65)	1			1		
	Yes, neighbor cat	108	38 (35.19)	0.73	0.20, 0.45	1.18	1.00	0.61, 1.64	1.00
	Yes, own cat	200	62 (31)	0.60	0.02, 0.40	0.91	1.31	0.87, 1.96	0.20
*C. burnetii* infection	Negative	127	28 (22.05)	1			1		
	Positive	96	45 (46.88)	1.61	0.78, 3.32	0.198	1.05	0.57, 1.95	0.840
*Brucella* Spp. infection	Negative	166	110 (66.27)	1					
	Positive	57	40 (70.18)	1.30	0.57, 2.96	0.531	1.86	0.9, 3.83	0.094
*C. abortus* infection	Negative	150	44 (29.33)	1			1		
	Positive	73	29 (39.73)	1.97	0.96, 4.04	0.066	1.04	0.55, 1.94	0.877
*T. gondii* infection	Negative	166	56 (33.73)	1			1		
	Positive	57	17 (29.82)	0.51	0.25, 1.04	0.063	1.22	0.66, 2.27	0.644
At least one infection	Negative	53	12 (22.64)	1					
	Positive	170	61 (35.88)	2.03	0.92, 4.51	0.081	2.23	1.03, 4.81	0.000
Mixed infection	Negative	124	33 (26.61)	1			1		
	Positive	99	40 (59.6)	1.49	0.78, 2.85	0.227	1.27	0.70, 2.3	0.591

*IRR, Incidence rate ratio; CI, Confidence interval*.

Nineteen variables with *p* ≤ 0.15 were included in the multivariable analysis, which retained seven variables in the final regression model. The result of the final zero-inflated negative binomial regression analysis is presented in Table 6. Agroecology and production system were controlled as a potential confounder in the model. Spending the night in traditional sheep houses' and “providing supplementary feed for pregnant dams” were important management factors which significantly (*p* ≤ 0.05) decreased the risk of abortion by 2.63 and 4.55 times, respectively. Presence of other livestock species and dogs in the household' had a marked effect on the risk of abortion in sheep and goat flocks. Moreover, exposure of the flock to *Brucella* spp. or anyone of the four tested infectious agents significantly (*p* ≤ 0.05) increased the risk of abortion in the flock by 1.66 and 1.68 times compared to non-exposed flocks, respectively.

**Table 6:** Final model of multivariable zero-inflated negative binomial regression analysis for effect of management and exposure to abortion pathogens on abortion in the flock.

**Variable**	**Category**	**IRR**	**95% CI**	***P*-value**
Agroecology and production system	Lowland MCL	1		
	Lowland Pastoral	0.53	0.34, 0.81	0.003
	Highland MCL	0.18	0.03, 1.06	0.058
Presence of other livestock	Small ruminant only			
	≤ 2 other livestock species	2.69	1.33, 5.42	0.006
	>2 other livestock species	2.22	1.15, 4.30	0.018
Housing/shelter at night	None shelter paddock	1		
	Traditional house	0.38	0.25, 0.58	0.000
Dog access to the flock	Not at all	1		
	Yes, neighbor dog	1.77	0.85, 3.67	0.127
	Yes, own dog	2.45	1.38, 4.37	0.002
Supplementary feed for pregnant	No	1		
	Yes	0.22	0.07, 0.70	0.01
*Brucella* Spp. *infection*	Negative	1		
	Positive	1.73	1.27, 2.36	0.001
At least one infection	Negative	1		
	Positive	1.85	1.38, 2.47	0.000

*IRR, incidence rate ratio; CI, Confidence interval*.

## Discussions

This study provided important insights on the occurrence, causes, and potential risk factors of abortion in small ruminant in smallholder systems in Ethiopia. The present study revealed that abortion is an important problem of small ruminant production in lowland mixed crop, livestock and pastoral agroecology and production system.

This study found overall annual abortion percentage of 16.1% in doe and 12.5% in ewe, which is in the range of previous reports from the mixed crop-livestock and pastoral production systems in Ethiopia, 9.3–40.9% for doe and 7.5–36.4% for ewes (31). Compared to the international figures, a higher abortion percentage of 43.7% for does and 35.6% for ewes was reported from Egypt (32). In contrary, a lower abortion rate has been reported from Jordan, 10.6% in does and 2.0% in ewes (33); from Nigerian, 10.8% in does (34) and from Mexico, 3.5% in does (35). Similarly, the higher abortion percentage in lowland flocks than in highland flocks is in agreement with the report of Gebremedhin et al. (22) and Fentie (31) in Ethiopia. Mixed crop-livestock and pastoral production systems are practiced in dryland agro-ecosystems where multiple stressors such as the cumulative effects of poor nutrition, excessive heat, and the need to walk long distances to source feed and water compromise the production and reproduction performance of small ruminants (36). The results from the household survey also indicated feed shortage as the second most important abortion cause in lowland pastoral and the third in lowland MCL agroecology and production system as perceived by livestock keepers. Lack of adequate year-round feed resources because of erratic rainfall could be the most important factor contributing to high reproductive failures such as abortion in arid and semiarid areas (37). Poor availability of quality feed in the drylands leads to low levels of energy during pregnancy, which markedly affects fetal survival, thus abortions and stillbirths are major causes of economic loss for the small ruminants managed in dryland areas under extensive management conditions (38, 39). Lower levels of glucose in the maternal blood due to nutritional stress trigger the hyperactivity of the adrenal glands of the fetus, which then releases the estrogenic precursors that leads to expulsion of the live fetus and, hence, abortion occurs (40). The monthly distribution of abortion incidence documented in this study clearly corresponds to the shortage of rain, which in turn affects the availability of feed in the study areas.

Furthermore, extreme ambient temperatures in lowland agroecology might contribute to the higher abortion rate in sheep and goats (41, 42). Heat stress in this agro-ecosystem leads to hyperthermia and may indirectly affect feed intake (43). Thermal stress during pregnancy is responsible for the abnormal development of the fetus due to impaired normal placental vascular development and less chance of survival as a result of compromised passive immunity (44, 45). The respondents from the lowland MCL agroecology and production system also highlighted the extreme weather conditions in May as the most important cause of sheep and goat abortion in the area.

Our findings revealed that abortion is widely prevalent in goat flocks compared to sheep flocks. The present study is consistent with previous studies in Ethiopia (22, 31, 33) and elsewhere (32). Almost all interviewed sheep and goat owners also confirmed the higher susceptibility of does to abortion than ewes. The reason for this might be genetical factors, physiological or higher susceptibility to risk factors present. In addition, higher infection rate of three of four tested abortion causing agents in goat might attributed for this higher number of abortions in doe than ewes. Moreover, the reproductive behavior of does is that they can tolerate moderate weight loss due to feed shortage at mating and still get pregnant. However, the fetuses might be maintained or expelled depending upon feed availability (46, 47).

The risk of abortion was significantly higher (*P* ≤ 0.05) in the small ruminant flocks which spent the night in non-shelter paddocks compared to the flock in the traditional shelters. In low-input extensive production systems, small ruminant flocks confined in non-shelter paddocks expose the animal to bad weather conditions, hence compromising the health and welfare status of the animal. Furthermore, in the majority of cases, households who have larger flock size confine their animals in non-shelter paddocks with insufficient area for resting of the animal, increased moisture, manure accumulation, and an overall decreased hygiene status which increase contact with pathogenic agents.

This study found that the presence of more than two livestock species in the household significantly increases the abortion risk in the flock. Since the majority of abortion causing agents are shared among livestock species (48), keeping more animal species at the household level may increase animal density and chance of contact between animals, thus facilitating cross-transmission between livestock species which increase the chance of acquiring infection. Another possible explanation for this is that livestock species such as cattle are considered the most important livestock species in lowland MCL and pastoral agroecosystem (5) and thus receive preferential treatment if resources are scarce resulting in low standard of management and inadequate feeding of sheep and goats.

Our findings revealed that the presence of dogs in the household could increase the risk of abortion significantly. Study in Algeria by Ghalmi et al. (49) also found the presence of dogs significant associated with the occurrence of abortion in cattle. One possible explanation for this is that dog might play a role in transmitting abortion causing agents to sheep and goat population which in turn increases the risk of abortion in the flocks (48, 50). Moreover, the dogs might be a mechanically spread infectious agents while feeding on infected placenta and aborted fetuses. Application of biosecurity precautions including burying or incinerating placentas and aborted fetuses helps to prevent the spread of the infectious organism and can reduce reproductive losses in sheep and goats (6). The presence of dogs in the household might increase the chance of chasing ewe/does by dogs. The stress of worrying by dogs can cause the pregnant dams to miscarry their lambs/kids (51).

This study also found that providing supplementary feed for dams in the last stage of pregnancy could significantly decreased the risk of abortion in the flock. Supplementary feeding will greatly depend on the availability and quality of forage. Where an animal is in an environment where feed resources are scarce, grazing needs to be supplemented with some level of concentrate feeding because the forage is not being balanced in terms of energy, protein, minerals, and vitamins (52). During the final stage of pregnancy, the fetus(es) can develop rapidly to acquire up to 75–80% of their future birth body weight (53). Hence, supplementing the dam with available feed resources in addition to grazing is important to fulfill the energy requirements of pregnant dams, and hence to increase pre- and post-natal survival of lambs and kids and birthweight and production for life.

Infectious causes of abortion play an important role in small ruminant abortion (7). Those pathogens are released into the environment through the aborted fetus, placenta, uterine fluids, and vaginal discharge of infected dams. These can serve as a source of infection for animal populations and cause zoonotic risks for farming communities (6, 48, 54). Serological analysis of serum might be useful for demonstrating evidence of exposure and estimating their role in sheep and goat abortion. The result of the present serological investigation indicated that all four infectious causes of abortion are widely distributed across three agro-ecologies and production systems might play an important role of in sheep and goat abortion. This study found a higher infection rate of *T. gondii* than other investigated abortion-causing agents. This might be related to the availability of favorable conditions for the maintenance and spread of this agent across the agroecology and production systems. The presence of both definitive hosts of *T. gondii* (cat) and intermediate hosts (rodents) in the area may influence the likelihood of contamination of feed, water, or pasture, which increased the risk for exposure of livestock to the parasite (55).

Our study found that exposure to *Brucella* spp. significantly increases the risk of abortion in sheep and goats, which is not surprising (56). The significant increment of the risk of abortion within the brucella infected flocks than across the flock might be due to the matter of fact that the incidence of brucella abortion in an already infected flock is low due to the development of herd immunity. However, there may be a high level of abortion in “abortion storm” in the newly infected flocks (1). Nevertheless, it did not find any significant association between the occurrence of abortion and infection of *C. burnetii, C. abortus* and *T. gondii*. This result agrees with the report from Gebremedhin et al. (22), Gebretensay et al. (15), and Tesfaye et al. (17) who reported that evidence of *C. burnetii, C. abortus*, and *T. gondii* infection did not associate with flock level abortion. However, many studies have demonstrated the role of *C. burnetti, C. abortus*, and *T. gondii* in sheep and goat abortion (48, 57). The absence of the association between those pathogens with sheep and goat abortion in this study might be related to maternal serum samples may yield positive results without the presence of abortion since the development of immunity within the flock prevents subsequent abortions (6, 48, 58, 59).

Moreover, the presence of at least one of the four infectious agents under investigation in the flock could significantly increase both the flock level of abortion prevalence and the number of abortions in the flock. This indicated the involvement of multiple infectious agents in sheep and goat abortion which put cumulative effects on small ruminant reproductive in different agroecology and production systems of Ethiopia. In agreement with the present findings, Bisias et al. (60), Mahboub et al. (61), and Benkirane et al. (62) reported the important role of multiple pathogens as causal agents of abortion in sheep and goat flocks. The results from the household survey also highlighted infectious diseases were the highest priority problem perceived and major concern for the producers as potential causes of sheep and goat abortion. This might correspond to an abortion incidence in June in lowland pastoral agroecology and production system in which grazing pasture is relatively good but still higher proportion of abortion in this agroecology and production system.

The role of management, agroecological and infectious disease factors on sheep and goat abortion was obvious in this study and our findings highlight the multifactorial nature of the problem. The findings also emphasize the potential for substantial improvement in reproductive loss from abortion by improving management and health practices that fit the respective agroecological zones. This requires integrated approaches that improve the nutritional state of pregnant dams through targeted supplementary feeding, abortion management through appropriate biosecurity practices, and vaccination programs for major infectious causes of abortion and herd health management through better veterinary services. To make sure interventions are sustainable and can be scaled, recognizing the farmer/pastoralist indigenous knowledge and participating them in the process as partners, creating an enabling environment to engage private sectors as service providers, building strong partnerships with key stakeholders and integrating other productivity improvement technologies are important strategies.

## Data Availability Statement

The original contributions presented in the study are included in the article/supplementary material, further inquiries can be directed to the corresponding author.

## Ethics Statement

The animal study was reviewed and approved by Addis Ababa University, College of Veterinary Medicine and Agriculture Animal Research Ethics Review Committee. Written informed consent for participation was not obtained from the owners because it was not required for this study in accordance with the national legislation and institutional requirements.

## Author Contributions

GA designed and implemented the study. BW and GM assisted with the study design. GA, BA, HD, AB, and MY conducted and monitored the data collection. BT and TB coordinated the laboratory analysis. GA analyzed the data and wrote the manuscript together with BW. All authors made contributions to the conception, design, and revision of the manuscript. All authors read and approved the final manuscript.

## Conflict of Interest

The authors declare that the research was conducted in the absence of any commercial or financial relationships that could be construed as a potential conflict of interest.
